# Is It Prime Time for Neutrophil Gelatinase-Associated Lipocalin?

**DOI:** 10.1016/j.ekir.2024.06.017

**Published:** 2024-06-12

**Authors:** Lui G. Forni, S. Sudha Mannemuddhu

**Affiliations:** 1 Royal Surrey Hospital, Guildford, Surrey, UK; 2 School of Medicine, University of Surrey, Guildford, UK; 3 East Tennessee Children's Hospital, University of Tennessee Medical Center, Knoxville, Tennessee, USA

See Clinical Research on Page 2443

“Prediction is very difficult, especially if it is about the future” (Neils Bohr)

There is a considerable body of evidence that demonstrates that the development of acute kidney injury (AKI), particularly stage 2 or 3, is associated with poor outcomes, both in the short-term and long-term, in adults and children.^1^ Currently, AKI is defined by changes in serum creatinine relative to a known or determined baseline value or specific changes in urine output. However, the Acute Disease Quality Initiative (ADQI) group have raised the construct that biomarkers of tubular stress and/or damage be considered as part of the classification of AKI stage rather than solely the classical functional markers.^2^ The logic behind this proposal is clear. Functional markers such as serum creatinine or indeed cystatin C, although providing the main analyte for chronic kidney disease, are of limited utility in predicting early kidney damage. Therefore, identification of candidate molecules which may identify injury early during the course of an episode is paramount, as this should allow for early intervention(s) which, in turn, may translate into improved outcomes. This has indeed been shown to be the case, with evidence that biomarker-guided interventions do in fact alter the course of and prognosis from AKI. In the PrevAKI study, the “KDIGO bundle” consisting of optimization of volume status and hemodynamics, avoidance of nephrotoxic drugs, and prevention of hyperglycemia was applied to patients undergoing cardiac surgery. These patients were deemed as high-risk, defined by a raised urinary biomarker ([TIMP-2]·[IGFBP7]).^3^ AKI was significantly reduced after intervention when compared with standard care. These results provide further evidence that the presence of a positive biomarker in the absence of the traditional functional markers achieving diagnostic threshold (described by some as “subclinical” AKI or as proposed by ADQI stage 1c) is associated with poorer outcomes. Similarly, in children, the TAKING FOCUS 2 study used a combination of urine neutrophil gelatinase-associated lipocalin (NGAL) and a risk score coupled with a continuous renal replacement therapy (CRRT) initiation goal at 10% to 15% weight-based fluid accumulation to optimize CRRT use. Compared to patients treated before this intervention, the median time from intensive care unit admission to CRRT initiation was 2 days shorter and ≥15% pre-CRRT fluid accumulation rate was lower. This resulted in both a reduced length of stay after CRRT discontinuation, and the total intensive care unit stay was also shorter. Unsurprisingly, this translated to a significant estimated saving of cost.^4^

In this article, Goldstein and colleagues present data from 2 multicenter, prospective landmark studies in critically ill children, in which the performance of a well-described biomarker, NGAL, was assessed.^5^ NGAL is a 25 kDa protein, which is considerably upregulated during tubular injury. NGAL has an extensive literature base supporting its use clinically, and at least 5 meta-analyses demonstrate that NGAL is a consistently good to excellent performer in terms of predicting AKI earlier than changes in functional markers in pediatric populations. The principal aim of the study was to produce an accepted reference standard cutoff concentration, which is needed in order to catalyze not only clinical adoption but also to satisfy clearance by the US Food and Drug Administration despite this significant back catalogue. One study acted as a derivation cohort (*n* = 257), which was subsequently validated in the other (*n* = 660), with the primary outcome being development of Kidney Disease Improving Global Outcomes stage 2 or 3 at 48 to 72 hours after admission to the intensive care unit. Eligibility criteria included mechanical ventilation, vasoactive medication use, history of solid organ transplantation, history of bone marrow or stem cell transplantation, or hypotension despite adequate volume resuscitation occurring within 24 hours of admission. Within this high-risk cohort, the authors achieved their aim, deriving an overarching urinary cut-off of 125 ng/ml with an area under the curve of the receiver operating characteristic of 0.83 (95% confidence interval: 0.77–0.9) with, importantly, a negative predictive value of 96.9% (95% confidence interval: 95.1–98.0).

So, now that a cut-off value is available, will this translate into clinical adoption of a biomarker which already boasts a long clinical track record? Before considering that one must question why widespread implementation of biomarkers associated with kidney damage has not already occurred. If we consider the available kidney biomarkers, there is a considerable body of evidence supporting their use, and yet this is often met with indifference at best and derision at worst in many quarters. Partly, this may be driven by the desire from parts of the nephrology community for the arrival of the perfect biomarker for AKI which of course, is a multifactorial syndrome defined by changes in insensitive functional markers. Given this caveat, the perfect biomarker almost certainly does not exist. This point cannot be stressed enough. Indeed, the often quoted “gold standard” clinically available biomarker, high sensitivity troponin T (used in a far more homogenous clinical scenario) is often used for its negative predictive ability.^6^ Many candidate molecules have been proposed as potential predictors for the development of AKI with variable performance.^7^ However, as shown by the authors, in this setting, NGAL not only performs excellently given the context in predicting the development of moderate to severe AKI, but it also has impressive negative predictor performance. So, why has the nephrology community failed to embrace the use of currently available biomarkers? Could this reflect confirmation bias *en masse*, where serum creatinine is seen as the only reliable marker of renal function and as such information supporting that premise is championed whereas evidence to the contrary is overlooked. Indeed, the initial clinical study on children undergoing cardiothoracic surgery was impressive in terms of predictive ability, and yet this is often ignored in favor of studies in adults with multiple comorbidities and heterogenous insults where, understandably, performance is less impressive.^8^

A biomarker widely used in medicine, the C-reactive protein is often employed in the critically ill to aid in the diagnosis of infection, and yet, despite daily measurements in many intensive care units worldwide, data are limited as to its role in affecting management or aiding diagnosis in isolation.^9^ Indeed, the adoption of C-reactive protein did not follow on from such intense statistical scrutiny and one wonders how it would have fared in today’s forensic arena. What makes the lack of adoption of NGAL even more curious is that the literature is currently awash with the application of artificial intelligence and machine learning methods to predict clinical outcomes using algorithms few of us fully understand, and yet these are embraced whereas the use of biomarkers with well-described pathophysiological roles are given significantly less credence. The authors should be commended for this study; hopefully, it will provide a call to arms to the pediatric nephrology community to embrace the use of NGAL rather than discard it. The time for nihilism has surely passed, and the widespread adoption of NGAL may herald in a new era of treatment for children at risk of AKI.

## Disclosure

LGF has received research funding from Baxter as well as speaker’s fees from Biomerieux, SphingoTec, and Baxter. The other author declared no competing interests.
